# Supplementary material for: Design and Evaluation of a Pediatric Resident Health Care Transition Curriculum
Source: MedEdPORTAL. 2022 Apr 1;18:11239. doi: 10.15766/mep_2374-8265.11239 (PMC8971142; doi:10.15766/mep_2374-8265.11239)
Supplement: Supplementary file 1 — Prerotation Test.docxPart 1.mp4Part 2.mp4Part 3.pptxPart 4.mp4Part 5.mp4Facilitator Guide.docxPostrotation Test.docxDidactic Module Evaluation.docxSummary Critique Evaluation.docx [file mep_2374-8265.11239-s001.zip › H. Postrotation Test.docx]

**HEALTH CARE TRANSITION DIDACTIC MODULE POST-TEST**

*(Please find the answer key on page 3.)*

1. Why is health care transition an important topic in primary care pediatrics?

1. One in five individuals between 12 and 17 years of age has a special health care need.
2. Ninety percent of children with chronic conditions survive into adulthood.
3. One half million youth with special health care needs become adults annually.
4. All of the above.

2. What is the goal of a well-planned health care transition?

1. To ensure a new primary care provider has been identified for a young adult.
2. To maximize lifelong functioning and well-being for all youth, including those who have special health care needs and those who do not.
3. To ensure that high-quality, developmentally appropriate health care services are available in an uninterrupted manner as the person moves from adolescence to adulthood.
4. Both B and C.

3. According to the 2016 National Survey of Children with Special Health Care Needs, what percentage of families of children and youth with special health care needs receive services for transition to adulthood?

1. 100%.
2. 80%.
3. 60%.
4. 40%.

4. Briefly describe, in your own words, the difference between “transition” and “transfer” of care.

5. According to the AAP Clinical Report on Health Care Transition, at what age should primary care providers begin discussing transition with families?

1. 10
2. 12
3. 14
4. 16
5. 21

6. Please list the 6 steps of the transition process for pediatricians described by the Got Transition initiative.

7. Which of the following is a strength a primary care provider may have on her team or in her toolbox to address the challenges of health care transition (check all that apply)?

1. Portable medical summaries.
2. Social work consult to address changes in insurance coverage.
3. Condition fact sheets.
4. Child life consult to address self advocacy.
5. Community-based supports and resources.
6. An electronic health record.
7. Medico-legal aid.

8. True/False: Under the provisions of the Affordable Care Act, young adults can stay on their parents’ insurance plan until the age of 21 years.

1. True.
2. False.

9. True/False: Upon review of the literature, primary care pediatricians often recognize the importance of a well-planned health care transition, but few are complying with all recommended steps.

1. True.
2. False.

10. If one is in place, please describe your institution's process for transitioning youth to an adult model of care.

11. Self management is defined as:

1. A set of behaviors that people engage in to balance their check book.
2. A set of behaviors that people engage in to pursue mindfulness.
3. A set of behaviors that people engage in as part of living with a chronic health condition.
4. A set of behaviors that people engage to balance time spent on numerous activities.

12. Please list three examples of self-management support (eg. attend to diet).

13. The gap between “capacity for” and “performance in” can help identify ________.

14. Health care transition resources for families with youth with special health care needs include:

1. Ronald McDonald Houses.
2. Got Transition.
3. State Parent to Parent Organizations.
4. Food Banks.

Answer Key

1. D

2. D

3. D

4. Transition is a well-planned process that addresses not only finding a new physician but also readiness, self-management, and appropriate compilation of medical records. Transfer is the identifying of a new provider and patient/family movement to this new provider.

5. B

6. Transition Policy, Tracking and Monitoring, Transition Readiness, Transition Planning, Transfer of Care, Transfer Completion.

7. All choices are correct.

8. F

9. T

10. [Answers will vary.]

11. C

12. [Answers will vary.]

13. Barriers.

14. B and C
