# Supplementary material for: Design and Evaluation of a Pediatric Resident Health Care Transition Curriculum
Source: MedEdPORTAL. 2022 Apr 1;18:11239. doi: 10.15766/mep_2374-8265.11239 (PMC8971142; doi:10.15766/mep_2374-8265.11239)
Supplement: Supplementary file 1 — Prerotation Test.docxPart 1.mp4Part 2.mp4Part 3.pptxPart 4.mp4Part 5.mp4Facilitator Guide.docxPostrotation Test.docxDidactic Module Evaluation.docxSummary Critique Evaluation.docx [file mep_2374-8265.11239-s001.zip › D. Part 3.pptx]

## Slide 1
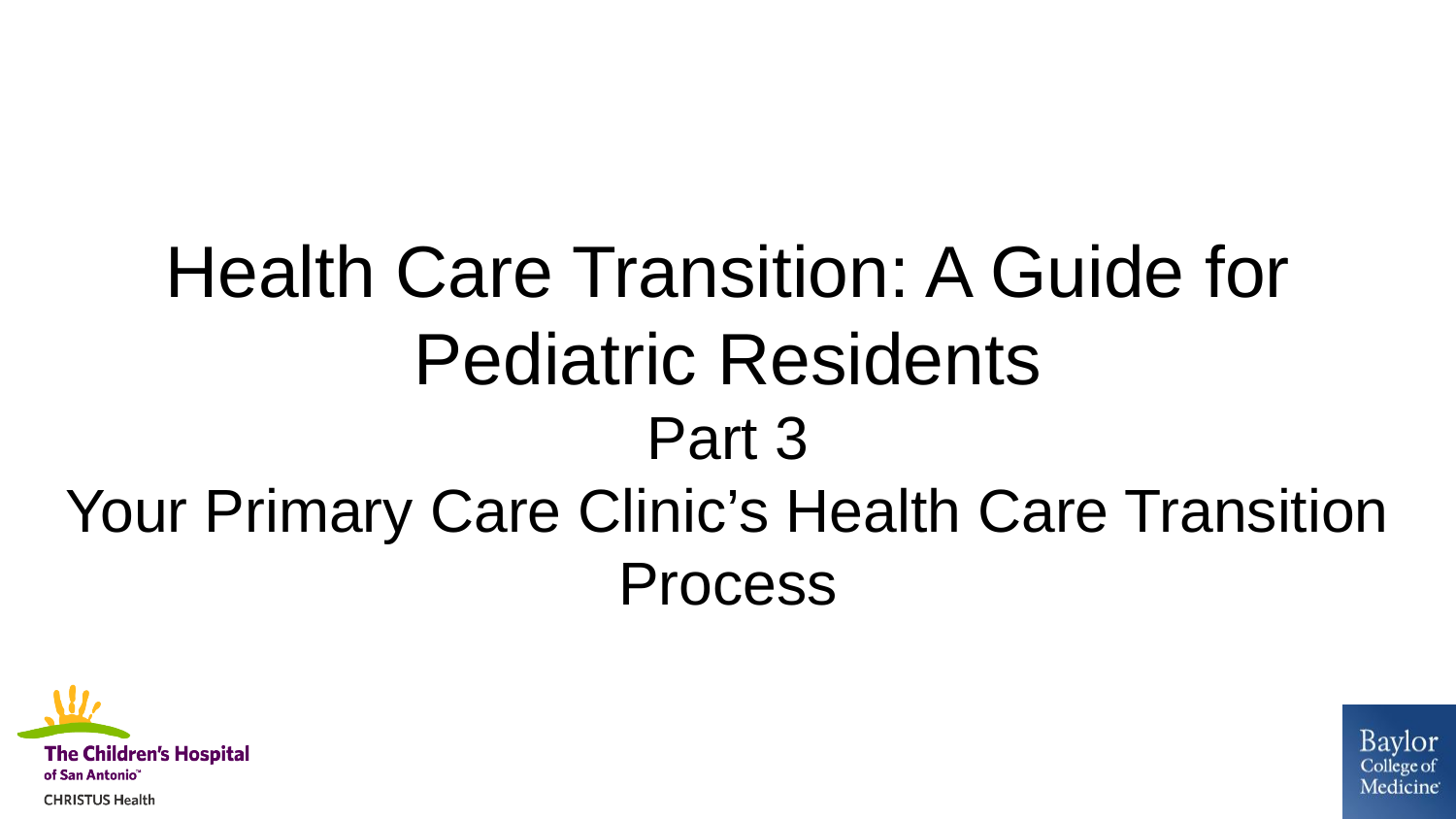

# Health Care Transition: A Guide for Pediatric Residents
Part 3
Your Primary Care Clinic’s Health Care Transition Process

## Slide 2
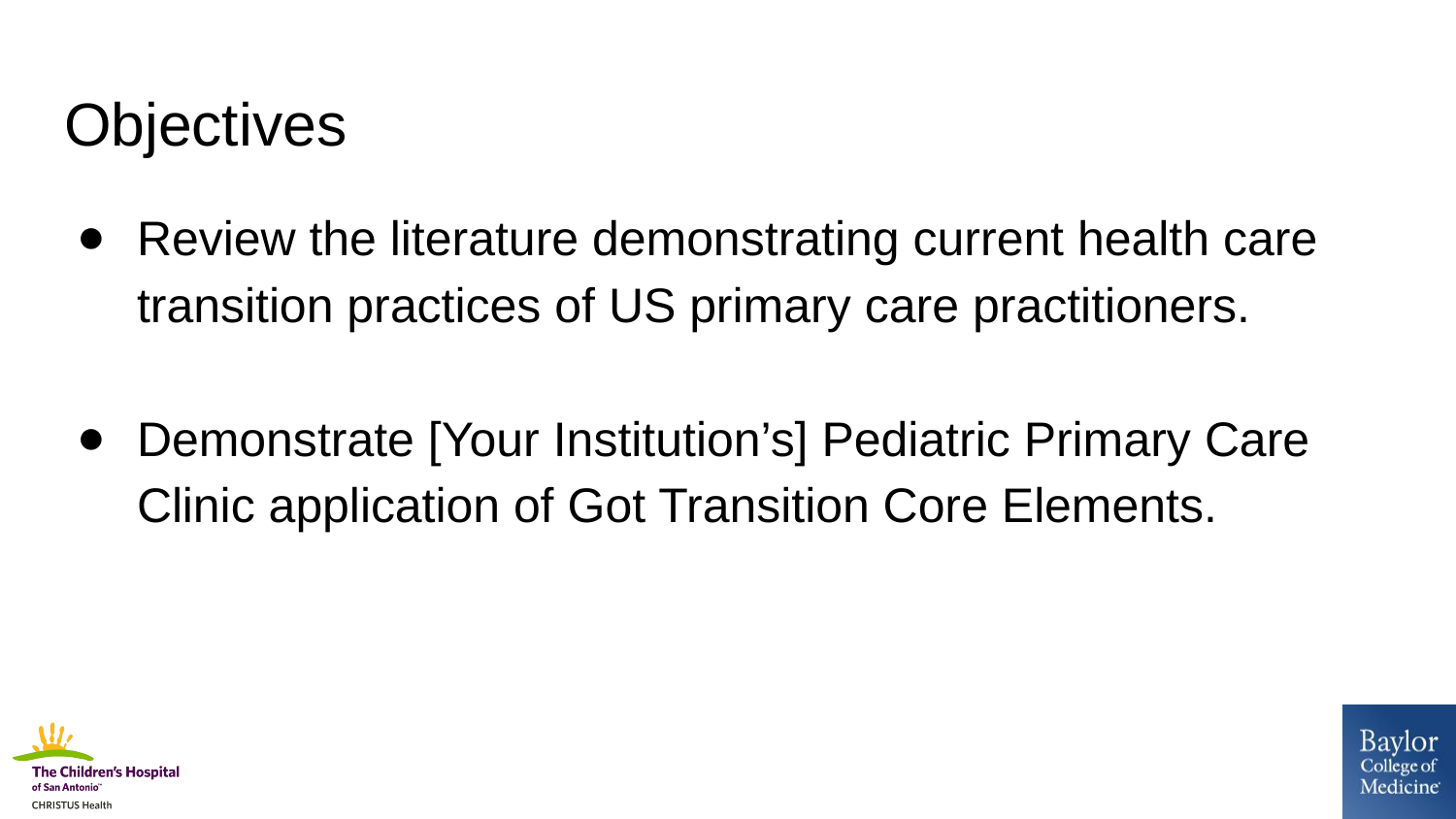

# Objectives
Review the literature demonstrating current health care transition practices of US primary care practitioners.
Demonstrate [Your Institution’s] Pediatric Primary Care Clinic application of Got Transition Core Elements.

## Slide 3
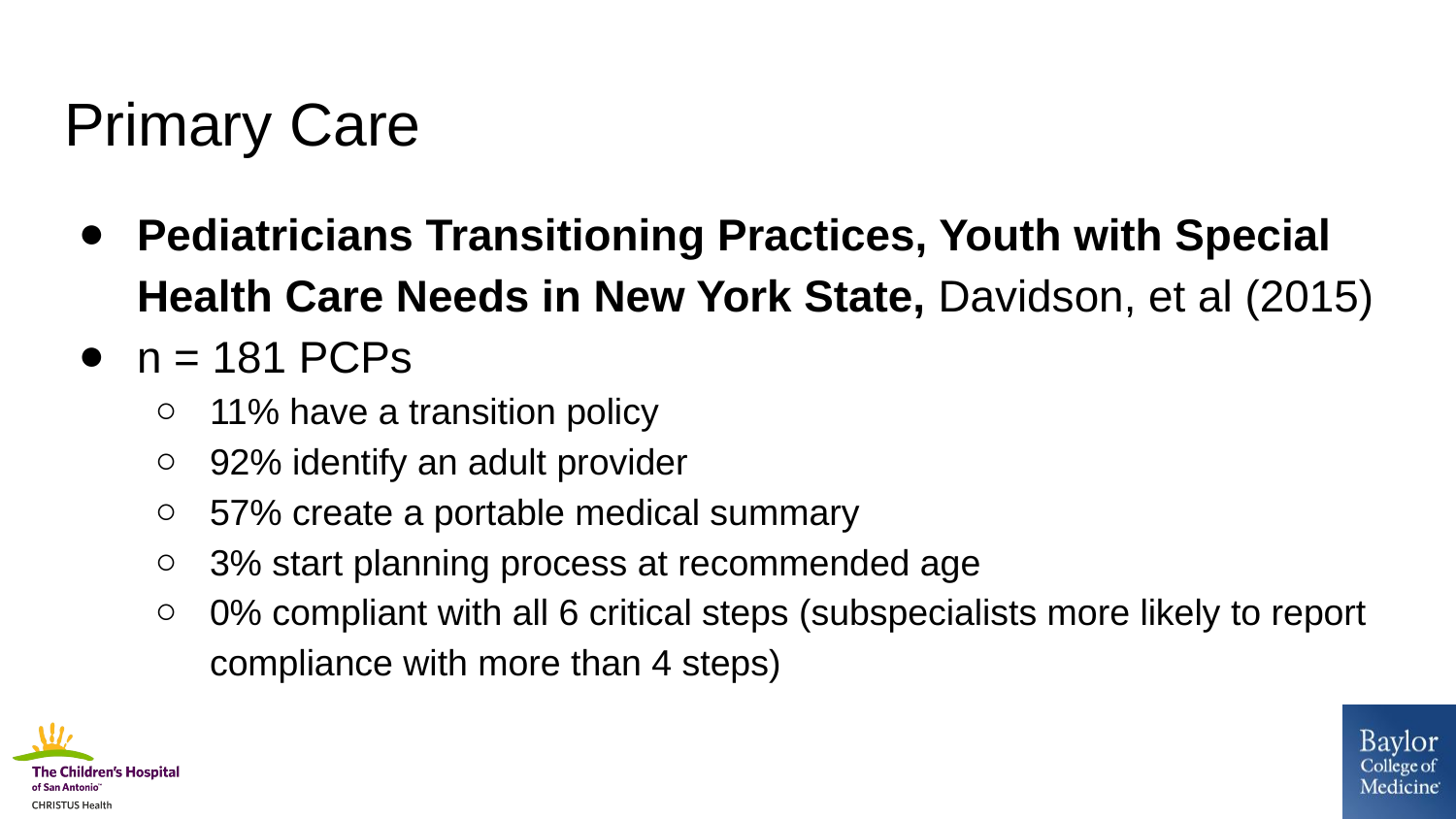

# Primary Care
Pediatricians Transitioning Practices, Youth with Special Health Care Needs in New York State, Davidson, et al (2015)
n = 181 PCPs
11% have a transition policy
92% identify an adult provider
57% create a portable medical summary
3% start planning process at recommended age
0% compliant with all 6 critical steps (subspecialists more likely to report compliance with more than 4 steps)

## Slide 4
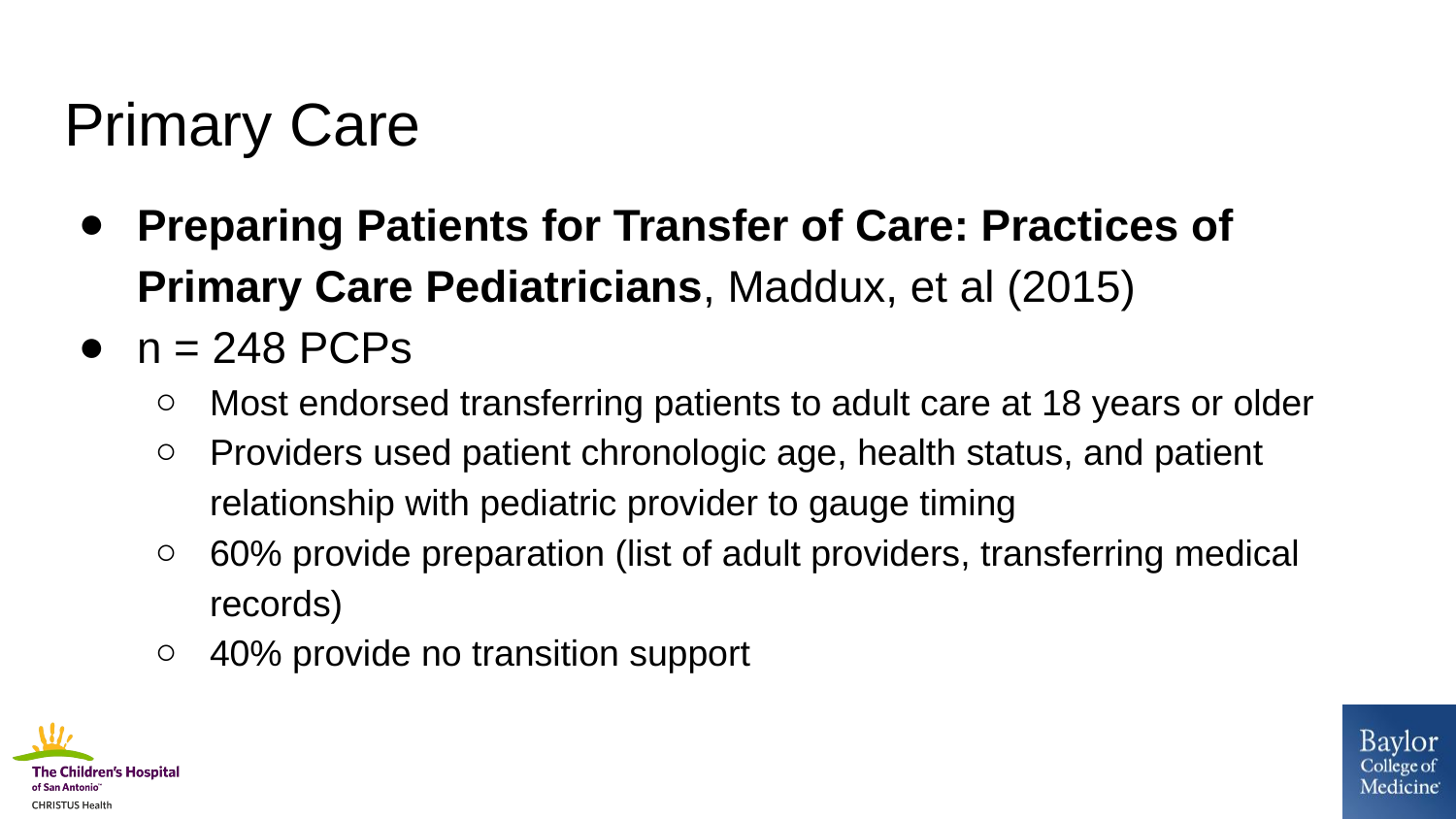

# Primary Care
Preparing Patients for Transfer of Care: Practices of Primary Care Pediatricians, Maddux, et al (2015)
n = 248 PCPs
Most endorsed transferring patients to adult care at 18 years or older
Providers used patient chronologic age, health status, and patient relationship with pediatric provider to gauge timing
60% provide preparation (list of adult providers, transferring medical records)
40% provide no transition support

## Slide 5
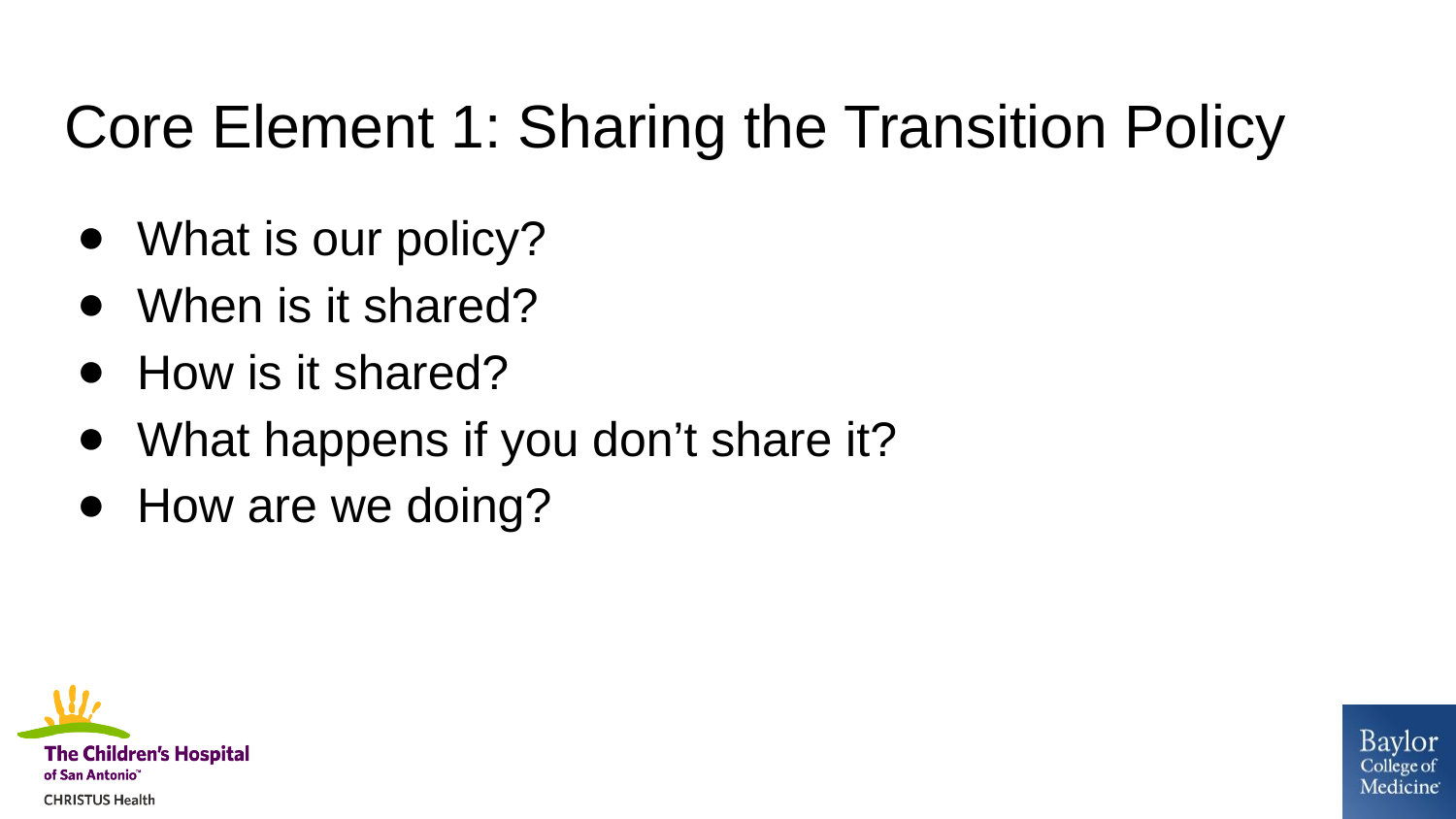

# Core Element 1: Sharing the Transition Policy
What is our policy?
When is it shared?
How is it shared?
What happens if you don’t share it?
How are we doing?

## Slide 6
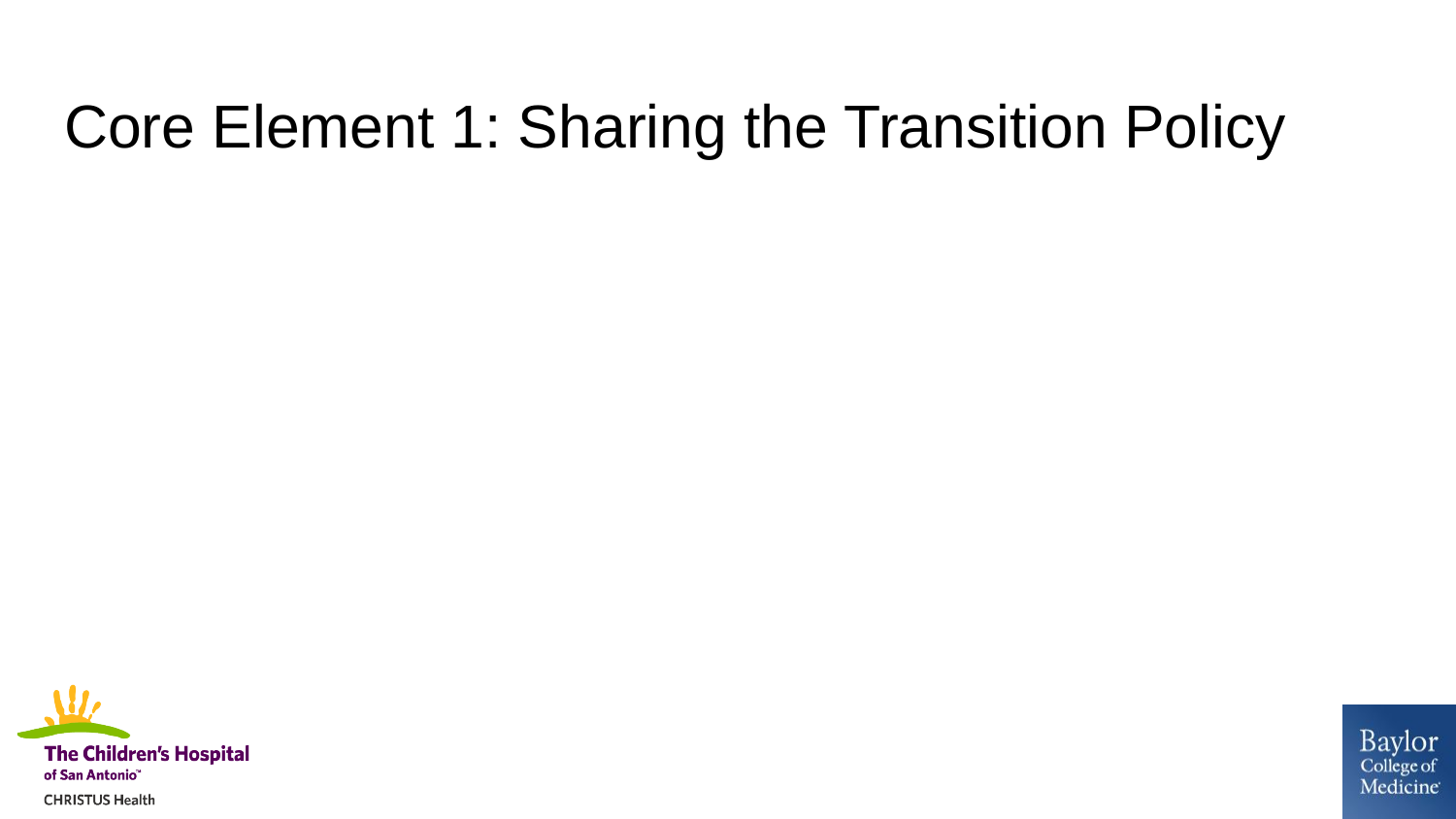

# Core Element 1: Sharing the Transition Policy

## Slide 7
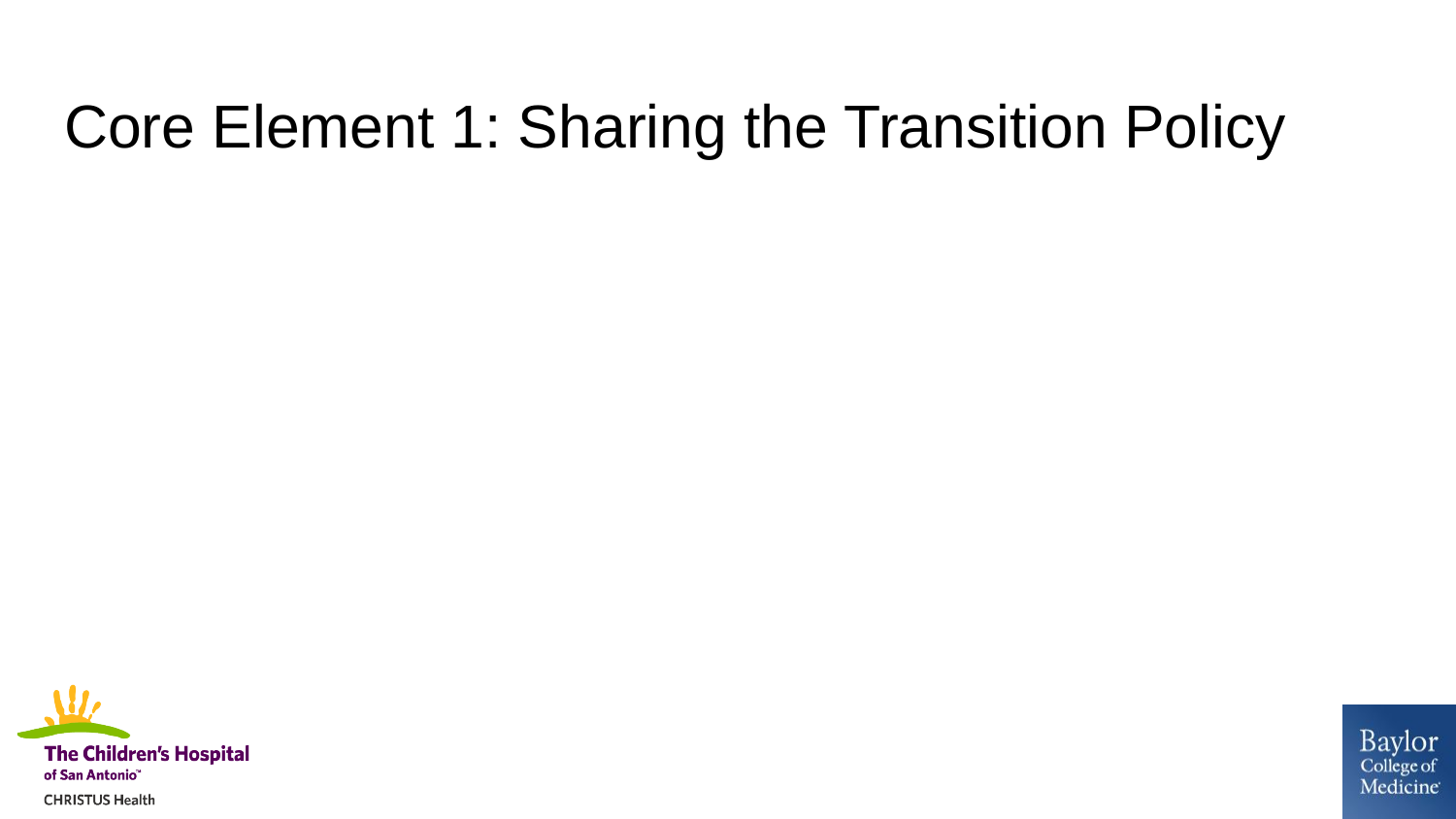

# Core Element 1: Sharing the Transition Policy

## Slide 8
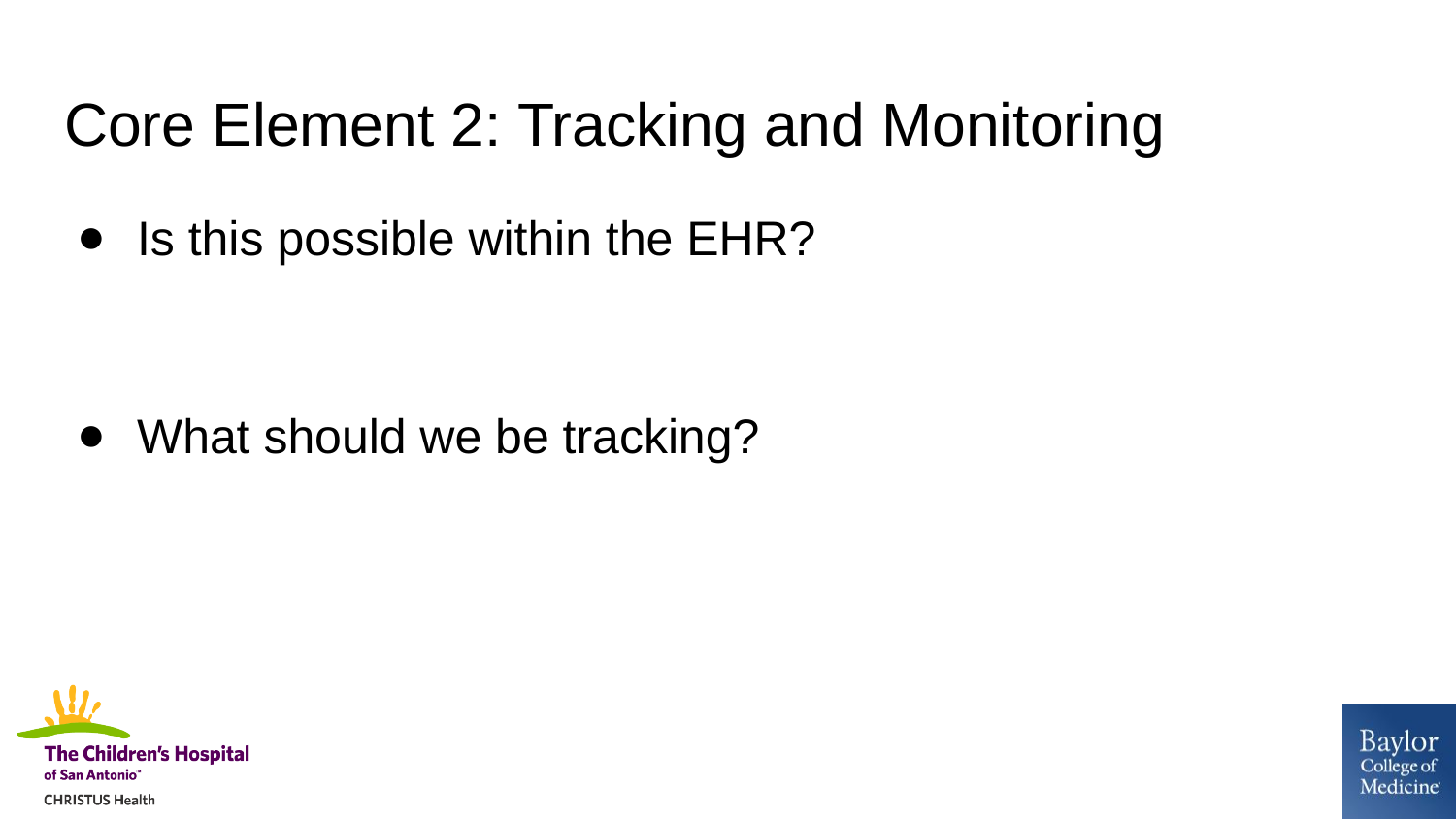

# Core Element 2: Tracking and Monitoring
Is this possible within the EHR?
What should we be tracking?

## Slide 9
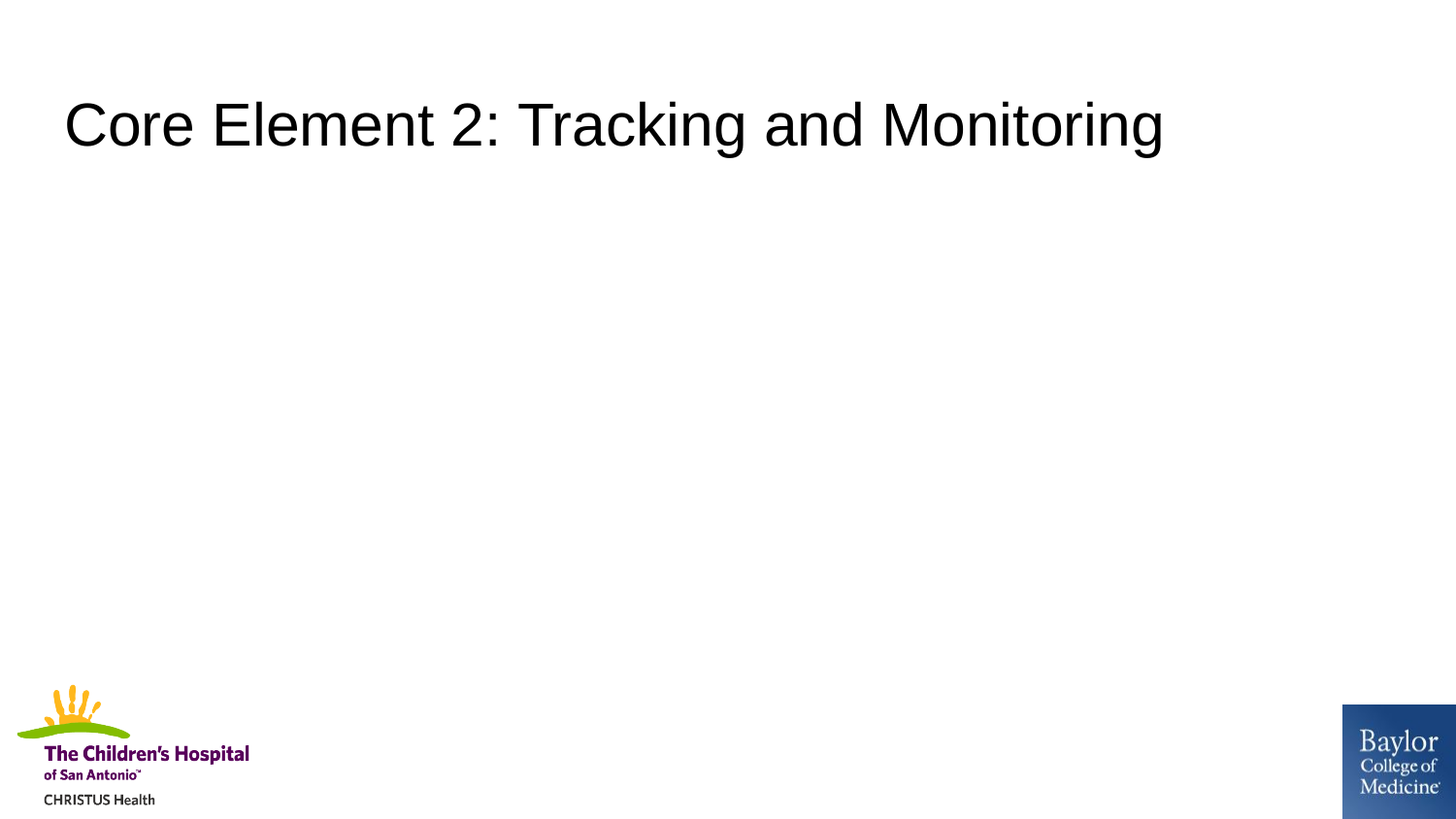

# Core Element 2: Tracking and Monitoring

## Slide 10
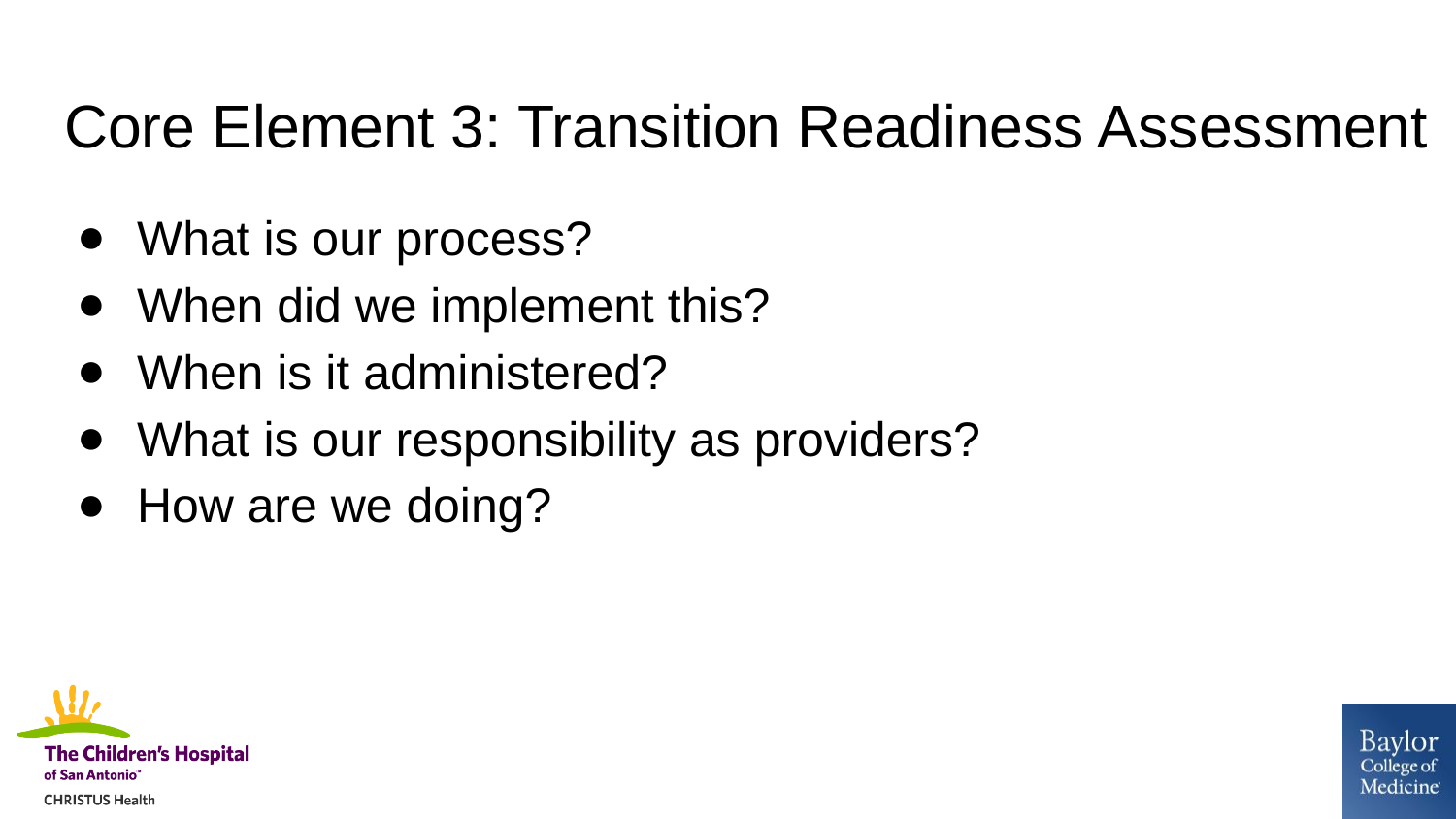

# Core Element 3: Transition Readiness Assessment
What is our process?
When did we implement this?
When is it administered?
What is our responsibility as providers?
How are we doing?

## Slide 11
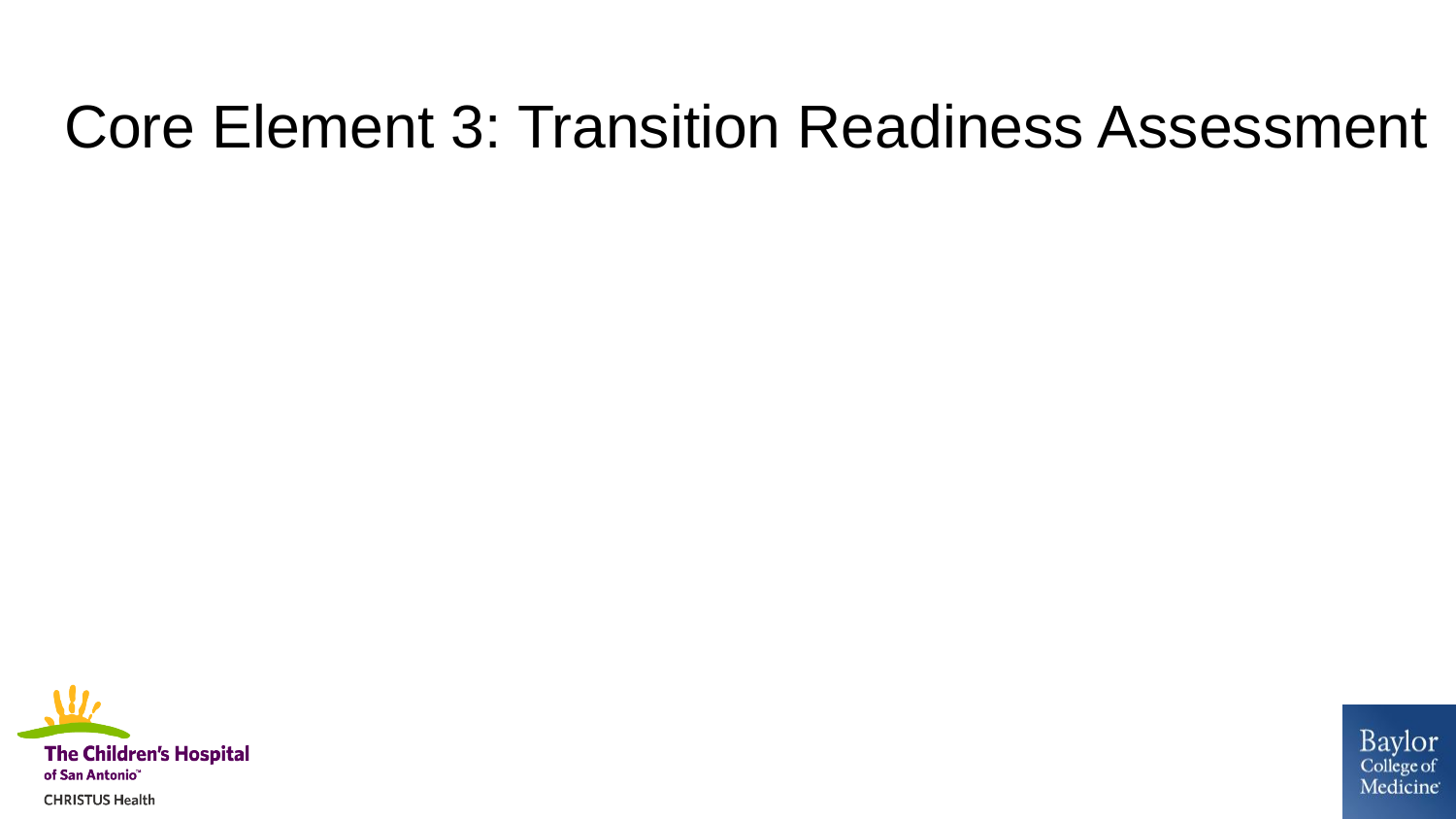

# Core Element 3: Transition Readiness Assessment

## Slide 12
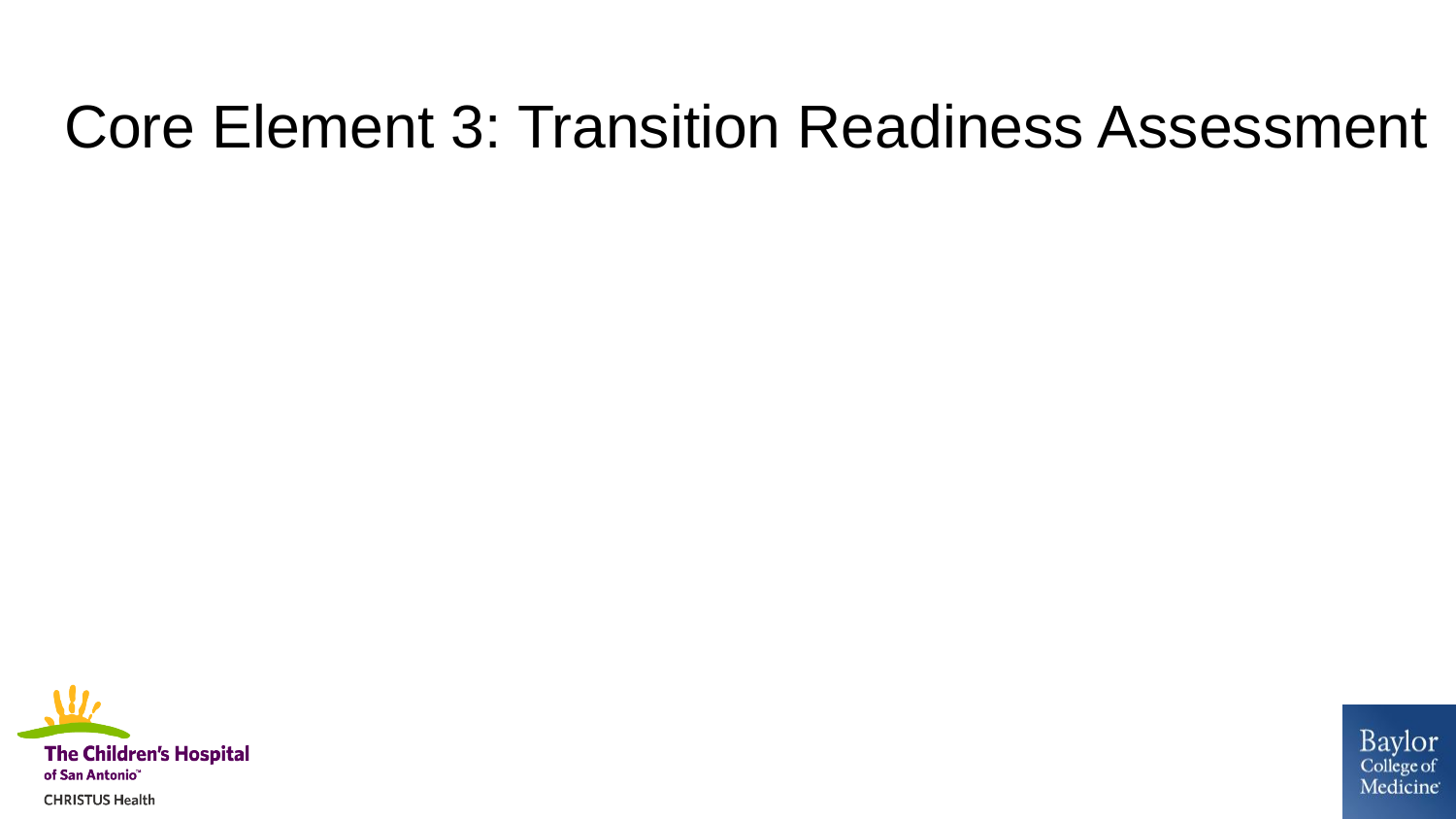

# Core Element 3: Transition Readiness Assessment

## Slide 13
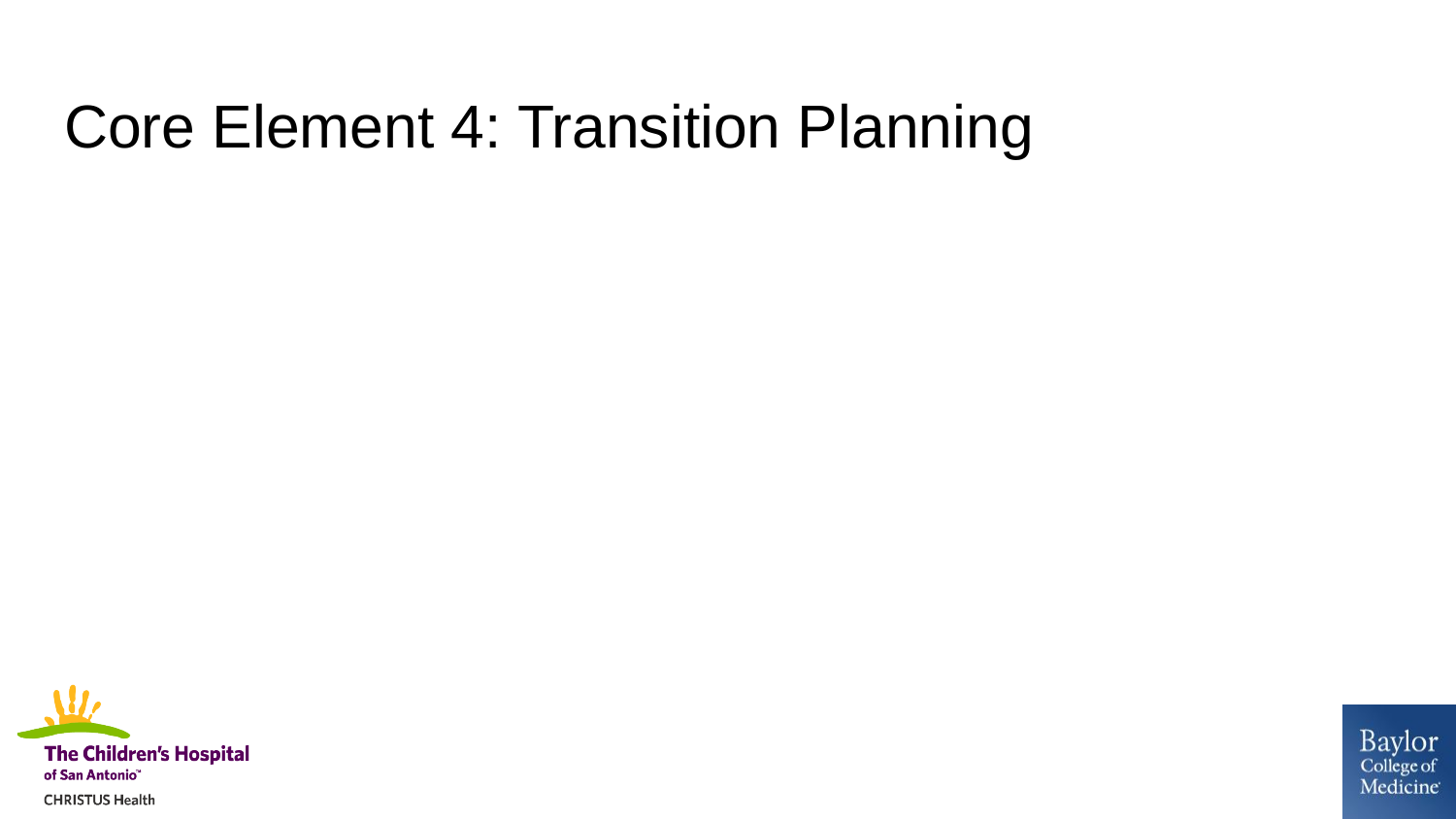

# Core Element 4: Transition Planning

## Slide 14
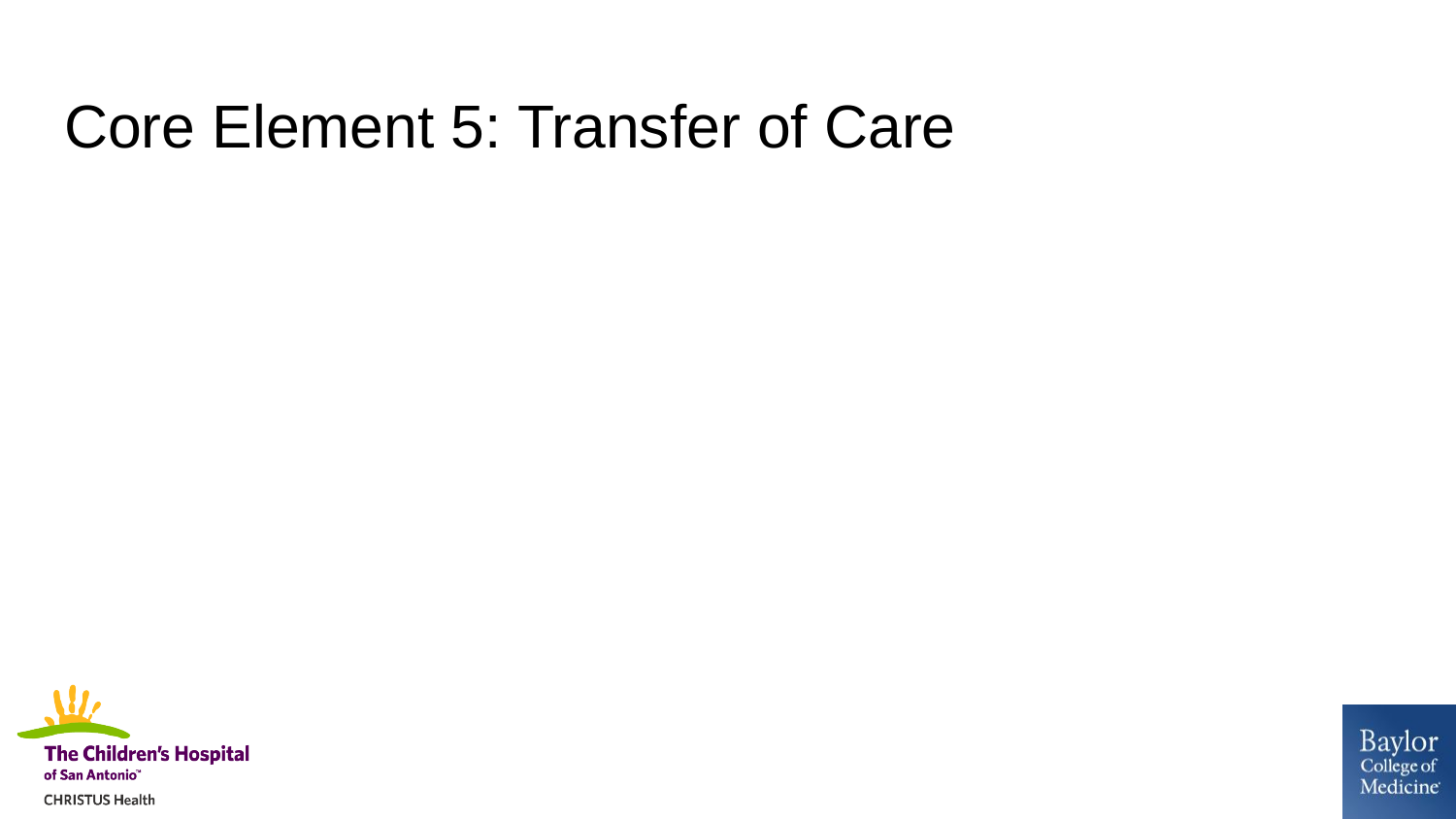

# Core Element 5: Transfer of Care

## Slide 15
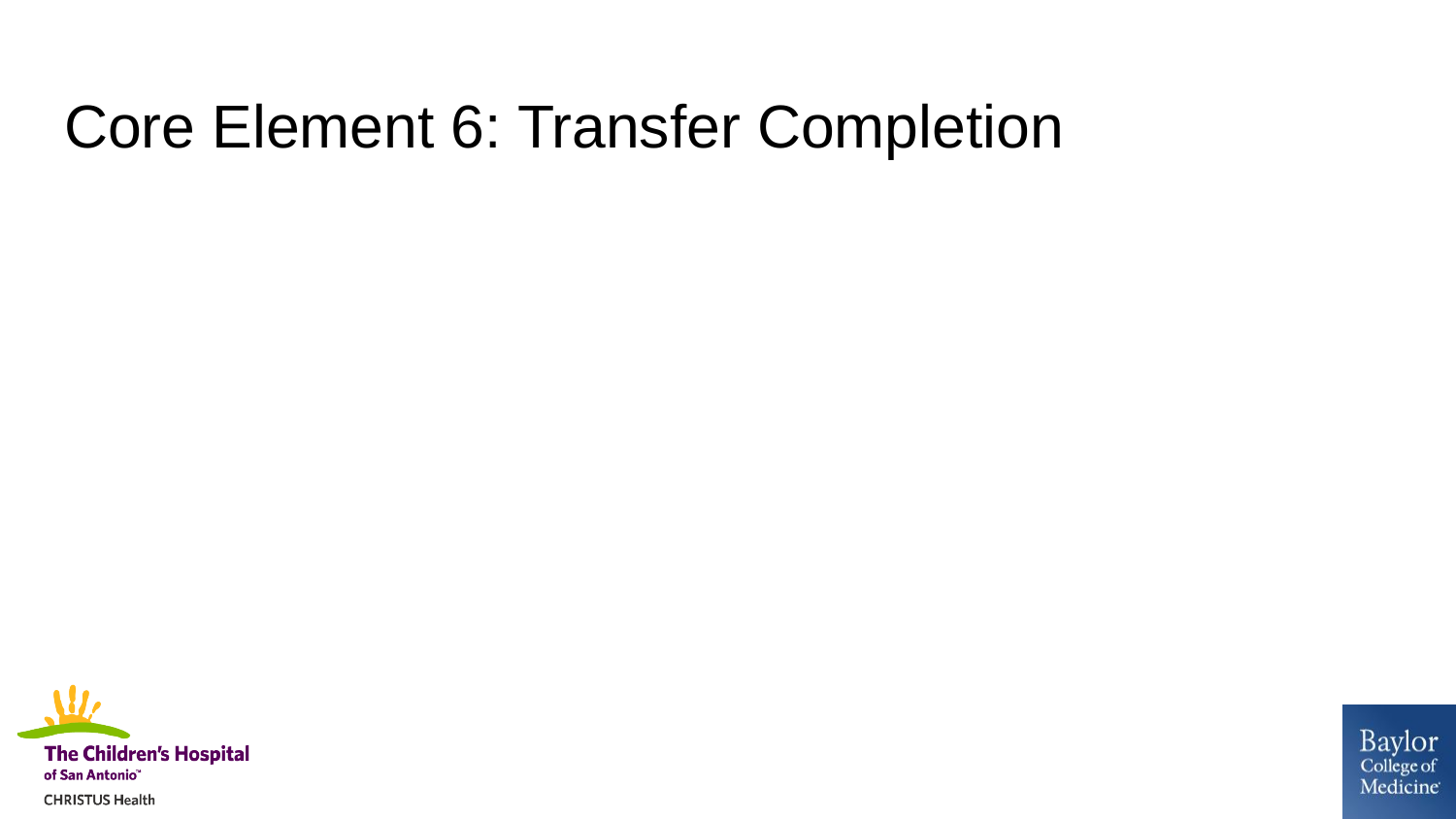

# Core Element 6: Transfer Completion
