# Supplementary material for: Design and Evaluation of a Pediatric Resident Health Care Transition Curriculum
Source: MedEdPORTAL. 2022 Apr 1;18:11239. doi: 10.15766/mep_2374-8265.11239 (PMC8971142; doi:10.15766/mep_2374-8265.11239)
Supplement: Supplementary file 1 — Prerotation Test.docxPart 1.mp4Part 2.mp4Part 3.pptxPart 4.mp4Part 5.mp4Facilitator Guide.docxPostrotation Test.docxDidactic Module Evaluation.docxSummary Critique Evaluation.docx [file mep_2374-8265.11239-s001.zip › G. Facilitator Guide.docx]

**Health Care Transition Facilitator’s Guide**

This facilitator’s guide will aid the educator in implementing Part 3 of the didactic modules and the Portable Medical Summary Critique Exercise.

**Part 3: Your Primary Care Clinic’s Transition Process**

Objectives

By the end of this activity, learners will be able to:

- Enumerate the steps of a well-planned health care transition (HCT) process and differentiate from transfer to an adult model of care.
- Implement a safe, well-planned HCT process utilizing established national resources within a primary care, medical home setting.

Target Audience

The target learners of this curriculum are pediatric residents. The target audience of this guide are educators who will deliver the curriculum to pediatric residents.

Setting

The Primary Care Clinic Health Care Transition Process facilitator’s guide may be used in ambulatory, continuity clinic, or complex care clinic settings.

Procedure

The facilitator’s guide is paired with the third *Health Care Transition: A Guide for Pediatric Residents* PowerPoint lecture. The guide describes the *initial* steps (Core Elements 1-4 of 6) taken to implement a HCT process within Baylor College of Medicine-The Children’s Hospital of San Antonio Pediatric Primary Care Clinic (BCM-CHofSA PPCC) utilizing resources obtained from the GotTransition ([www.gottransition.org](http://www.gottransition.org)) webpage.

- **Core Element 1 -- Sharing the Transition Policy**: Prior to 2018 the BCM-CHofSA PPCC did not have a formal process to discuss HCT with patients/families. We adapted the GotTransition Sample Transition Policy to align with our clinic’s procedures. The PPCC opted to begin sharing the PPCC Transition Policy (PPCC TP) with patients/families beginning at the 14 year well child care visit and every well child visit thereafter. Initially we asked front desk staff to distribute the PPCC TP when patients/families checked in for the visit. We discovered that we were unable to track whether patients/families were truly receiving the policy and that distribution of the policy by non-clinical staff failed to prompt a conversation regarding HCT between healthcare practitioners and patients/families. Consequently, we collaborated with our electronic health record (EHR) team members to incorporate the PPCC TP, in both English and Spanish, within the EHR. The policy was added as an “order” to auto-populate with “order sets” for well child care visits for patients > 14 years. Following four Plan-Do-Study-Act cycles, 91% of all well child care visit encounter notes for patients > 14 years demonstrated documentation of sharing of the PPCC TP and almost one in four visit encounter notes revealed documentation of a HCT discussion between healthcare practitioners and patients/families.*
- **Core Element 2 -- Tracking and Monitoring**: Tracking and monitoring vary across EHRs. We encourage educators to discuss options for tracking and monitoring the HCT process with their EHR team members. Tracking and monitoring proved challenging with our EHR. Using the GotTransition Sample Individual Flow Sheet as a template, we created a dot phrase (.ppcc_tra) to auto-populate the elements of this flow sheet within the Discussion Notes portion of the visit encounter note. The Discussion Notes are included in the Patient Visit Summary that is printed and handed to or published to the patient portal following the visit. One limitation of this method is that the Discussion Notes do not “pull forward” and must be rewritten for every visit encounter note.

.ppcc_tra

**Transition to Adult Care Tracking/Monitoring**

Transition Policy shared date:

Transition Survey completed date:

Patient or Parent?:

Supported decision-making:

Current insurance coverage:

Adult insurance coverage:

Medical Summary/Emergency Care Plan created date:

Name of new Adult Provider:

Date of first appointment:

Date of communication with new Adult Provider:

Notes:

- **Core Element 3 -- Transition Readiness**: Prior to 2018 we did not have a formal process to assess patient/family HCT readiness. We began by using the GotTransition Sample Transition Readiness Assessment at all well child care visits > 14 years. After limited success, upon discussion we discovered that PPCC faculty found the GotTransition Sample Transition Readiness Assessments lengthy for our patients/families and that PPCC faculty believed 14 to be too early to begin assessment of transition readiness. We worked with our Child Life department and Family Advisory Board to draft a more concise Transition Survey and began to ask patients to complete at well child care visits > 16 years. For patients with intellectual disabilities, we asked caregivers to complete the survey. Nursing staff presented surveys to patients/families while rooming patients, and healthcare practitioners reviewed responses and addressed gaps during the visit. Following four Plan-Do-Study-Act cycles, 48.4% of well child care visit encounter notes for patients > 16 years demonstrated documentation of a completed Transition Survey (scanned into the EHR, attached to the visit encounter note).*
- **Core Element 4 – Transition Planning**: A full description of our HCT planning processes is outside of the scope of this guide, as transition planning is complex and entails numerous considerations. We limit the narration of our transition planning process to the development of the portable medical summary (PMS). Again, we used the GotTransition Sample Medical Summary and Emergency Care Plan as a template to create a PMS (see below), including 18 vital elements to best outline the essential pieces of the medical record for an accepting adult healthcare practitioner. Many EHRs will allow such a summary to be an easily locatable living document that can be edited at each subsequent visit. Our EHR does not have this capability, and, as such, we used the PMS to create a “Letter” type. The Letter must be generated at every visit or a previous Letter can be found, reopened, edited, and closed. Once closed the Letter may be printed and handed to the patient/family or published to the patient portal. Each Letter has a document ID that we opted to add to the “Alerts” at the top of the chart to allow us to easily locate it.

*Outcomes of these QI projects were accepted for poster presentation at the 2019 Health Care Transition Research Consortium in Houston, Texas.

**Portable Medical Summary Critique Exercise Facilitator's Guide**

Objectives

By the end of this activity, learners will be able to:

- Enumerate the elements of a well-written portable medical summary (PMS).
- Explain the clinical significance of various elements of the PMS.
- Apply this knowledge by creating a PMS for a patient with a chronic medical condition.

Target Audience

The target learners of this activity are pediatric residents. The target audience of this guide are educators who will deliver the curriculum to pediatric residents.

Setting

This Portable Medical Summary Critique Exercise facilitator’s guide may be used in ambulatory, continuity clinic, or complex care clinic settings.

Procedure

This activity is paired with and serves as the learner assessment for the Part 5 video lecture.

1. Instruct the learner to identify a patient with a chronic medical condition.
2. Ask the learner to create a PMS consisting of the elements they believe should be included. Ideally, the learner will complete this without a search for resources.
3. Review the learner’s PMS and discuss the elements of a well-written PMS enumerated on the following page. This list was adapted from the GotTransition ([www.gottransition.org](http://www.gottransition.org)) sample medical summary.
4. If your electronic health record (EHR) allows, consider designing a PMS that will auto-populate all of these elements within the EHR and demonstrate for the resident.
5. Ask the learner to recreate the PMS for the same patient, within the EHR if possible, and review for completeness.

Portable Medical Summary Elements

**Learner Name / PGY** (This information is for the faculty preceptor’s records.)

**Date of Completion** (This information is for the faculty preceptor’s records.)

**Two-Step ID** (Patient Name, DOB)

**BEST Contact Info**: The learner may need to collect this information during a patient encounter as contact information on clinic demographic forms is not always the BEST contact information.

**Emergency Planning**: This may include preferred hospital, contact information of providers in order of priority, and quickly accessible orders in case of emergency (eg. glucagon order in case of hypoglycemia).

**Medical Decision-Maker**: For individuals 18 years and older, legal determination is required if the medical decision-maker is someone other than the patient.

**Code Status**

**Baseline Neurologic Status**: This information is particularly helpful for new adult providers and inpatient health care professionals who have not yet become familiar with the patient.

**Allergies**

**Medications**

**Problem List**

**Past Medical History**

**Past Surgical History**

**Social History**

**Private Duty Nursing, Habilitative/Rehabilitative Therapy, Durable Medical Equipment Vendors**

**School**: This includes plans for completion of high school, vocational training, and/or higher education.

**Provider List**

**Immunization Record**

**Most Recent Vital Signs**

**Most Recent Assessment / Plan**
