# Supplementary material for: Design and Evaluation of a Pediatric Resident Health Care Transition Curriculum
Source: MedEdPORTAL. 2022 Apr 1;18:11239. doi: 10.15766/mep_2374-8265.11239 (PMC8971142; doi:10.15766/mep_2374-8265.11239)
Supplement: Supplementary file 1 — Prerotation Test.docxPart 1.mp4Part 2.mp4Part 3.pptxPart 4.mp4Part 5.mp4Facilitator Guide.docxPostrotation Test.docxDidactic Module Evaluation.docxSummary Critique Evaluation.docx [file mep_2374-8265.11239-s001.zip › I. Didactic Module Evaluation.docx]

**DIDACTIC MODULE EVALUATION**

1. Have you viewed all five "Health Care Transition: A Guide for Pediatric Residents" didactic modules?.

Yes

No

2. How effective were the didactic modules in presenting you with new information?

| Not at all effective | Somewhat effective | Moderately effective | Very effective | Extremely effective |
| --- | --- | --- | --- | --- |

3. How engaging were the didactic modules?

| Not engaging at all | Somewhat engaging | Moderately engaging | Very engaging | Extremely engaging |
| --- | --- | --- | --- | --- |

4. How appropriate was the format of the didactic modules for learning this topic?

| Not appropriate at All | Somewhat appropriate | Moderately appropriate | Very appropriate | Extremely appropriate |
| --- | --- | --- | --- | --- |

5. How ideal was the format of the didactic modules for your schedule?

| Not ideal at all | Somewhat ideal | Moderately ideal | Very ideal | Extremely ideal |
| --- | --- | --- | --- | --- |

6. How likely are you to make changes to your health care transition practices after viewing the didactic modules?

| Not at all likely | Somewhat likely | Moderately  likely | Very likely | Extremely likely |
| --- | --- | --- | --- | --- |

7. What are some strengths of this health care transition curriculum?

8. What are some opportunities for this health care transition curriculum?

9. Do you have any other comments about this health care transition curriculum?
