# Supplementary material for: Design and Evaluation of a Pediatric Resident Health Care Transition Curriculum
Source: MedEdPORTAL. 2022 Apr 1;18:11239. doi: 10.15766/mep_2374-8265.11239 (PMC8971142; doi:10.15766/mep_2374-8265.11239)
Supplement: Supplementary file 1 — Prerotation Test.docxPart 1.mp4Part 2.mp4Part 3.pptxPart 4.mp4Part 5.mp4Facilitator Guide.docxPostrotation Test.docxDidactic Module Evaluation.docxSummary Critique Evaluation.docx [file mep_2374-8265.11239-s001.zip › J. Summary Critique Evaluation.docx]

**POST-MEDICAL SUMMARY CRITIQUE EVALUATION**

1. Prior to **reading the elements of a well-written medical summary**, how confident were you in your ability to create a well-written portable medical summary, including most of the elements listed in a well-written portable medical summary?

| Not at all confident | Somewhat confident | Moderately confident | Very confident | Extremely confident |
| --- | --- | --- | --- | --- |

2. Prior to **completing the critique with [Educator’s Name Here]**, how confident were you in your ability to create a well-written portable medical summary, including most of the elements listed in a well-written portable medical summary?

| Not at all confident | Somewhat confident | Moderately confident | Very confident | Extremely confident |
| --- | --- | --- | --- | --- |

3. How appropriate was the **format of learning** how to compose a well-written portable medical summary (writing a medical summary, reading a well-written summary, [Educator’s Name Here] critique of your portable medical summary) for this skill?

| Not at all appropriate | Somewhat appropriate | Moderately appropriate | Very appropriate | Extremely appropriate |
| --- | --- | --- | --- | --- |

4. How **likely are you to make changes** to future portable medical summaries after participating in this critique?

| Not at all likely | Somewhat likely | Moderately likely | Very likely | Extremely likely |
| --- | --- | --- | --- | --- |

5. How confident are you in your ability to compose a well-written portable medical summary after participating in this critique?

| Not at all confident | Somewhat confident | Moderately confident | Very confident | Extremely confident |
| --- | --- | --- | --- | --- |
