# Supplementary material for: Design and Evaluation of a Pediatric Resident Health Care Transition Curriculum
Source: MedEdPORTAL. 2022 Apr 1;18:11239. doi: 10.15766/mep_2374-8265.11239 (PMC8971142; doi:10.15766/mep_2374-8265.11239)
Supplement: Supplementary file 1 — Prerotation Test.docxPart 1.mp4Part 2.mp4Part 3.pptxPart 4.mp4Part 5.mp4Facilitator Guide.docxPostrotation Test.docxDidactic Module Evaluation.docxSummary Critique Evaluation.docx [file mep_2374-8265.11239-s001.zip › _Educational_Summary_Report_11239.pdf]

MedEdPORTAL® | The AAMC Journal of Teaching and Learning Resources

Original Publication Open Access

Design and Evaluation of a Pediatric Resident Health Care Transition Curriculum

Ruchi Kaushik, MD, MPH*, Virginia Niebuhr, PhD

*Corresponding author: complexcarepedi@gmail.com

Abstract

Introduction: In 2011, the American Academy of Pediatrics developed a consensus statement urging physicians who provide care to youth with special health care needs to acquire the knowledge and skills to facilitate well-timed transitions to adult-oriented care. However, a minority of these youth receive the services necessary to make appropriate transitions. Two potential barriers to supporting well-planned transitions are minimal provider training and gaps in medical records. **Methods:** We designed an adaptable health care transition (HCT) curriculum combinings asynchronous didactic modules and a synchronous portable medical summary (PMS) critique exercise to improve resident knowledge, skills, and behavior. Residents completed pre- and posttests to assess knowledge prior to and after viewing animated video didactic modules. Residents attempted to create a PMS, received feedback and instruction on how to create a well-written PMS, and then reattempted this activity. Residents evaluated both the didactic modules and the PMS critique exercise following delivery of the curriculum. **Results:** Over 21 months, 20 pediatric residents and hospital medicine fellows completed the curriculum during an elective complex care block rotation. Pre- and posttests revealed statistically significant ($p < .001$) improvement in knowledge. Learners included an average of 46% of 18 recommended PMS elements before and 98% of elements after the PMS critique exercise ($p < .001$). Evaluations demonstrated overwhelmingly positive learner responses. **Discussion:** Our adaptable HCT curriculum improves pediatric residents' knowledge, skills, and behavior in transition processes and addresses a significant gap in pediatric graduate medical education.

Keywords
Health Care Transition, Children With Medical Complexity, Pediatrics

Educational Objectives

By the end of this activity, learners will be able to:

1. Recognize the current state of health care transition (HCT) in the US and the numerous aspects to consider when planning seamless HCTs.
2. Enumerate the steps of a well-planned HCT process.
3. Implement a well-planned HCT process.
4. Support youth with special health care needs (YSHCN) with self-management when planning an HCT process.
5. Identify local and state community-based resources that support the HCTs of YSHCN and their families.
6. Apply established HCT guidelines to the development of portable medical summaries within the electronic health record.

Citation:
Kaushik R, Niebuhr. Design and evaluation of a pediatric resident health care transition curriculum. *MedEdPORTAL*. 2022;18:11239.
https://doi.org/10.15766/mep_2374-8265.11239

Introduction

In 2011, the American Academy of Pediatrics developed a consensus statement urging physicians who provide care to youth with special health care needs (YSHCN) to acquire the knowledge and skills to facilitate well-timed transitions to adult-oriented health care.[1] However, there is evidence that a mere 40% of YSHCN receive the services necessary to make appropriate transitions to adult-oriented care.[2]

The numerous barriers to an effective health care transition (HCT) include parent/caregiver concerns for youth's ability to self-manage,[3] adult providers' lack of comfort in managing pediatric conditions,[3] limited sharing of relevant medical records and health information between pediatric and adult health care providers,[4] and inadequate training in effective HCT practices for emerging pediatricians.[5]

Self-management support for adults is associated with improved health outcomes and patient experience, and evidence-based best practices and care models to inform self-management

Copyright © 2022 Kaushik and Niebuhr. This is an open-access publication distributed under the terms of the Creative Commons Attribution license.

support exist for the care of adults with chronic conditions.[6] These same practices may not be as common for YSHCN making the pediatric-to-adult transition. In a study describing mothers' experiences of transitioning their youth with type 1 diabetes mellitus, Ness and colleagues revealed mothers' heightened concern during the college transition compared to families of youth without diabetes and concluded that increasing preparedness for diabetes self-management would serve to decrease mothers' stress.[7] In 2018, Lozano and Houtrow outlined models for supporting self-management among young adults, including processes to address modifiable influences at the individual, family, community, and health care system levels.[6] Gaps in self-management support, however, remain.[8]

Gaps in the medical records often contribute to adult providers' lack of comfort with managing patients transferring into their care. Portable medical summaries (PMSs) are concise documents detailing relevant health information for YSHCN to prevent disruptions in care[4] and serve as a guide for patients/families and adult providers establishing new health care relationships with YSHCN.[9,10] Adult providers accepting YSHCN have expressed the need for a PMS prior to the first outpatient visit.[11-13] Nevertheless, in a sample of 181 primary care pediatricians, although 92% identified adult providers to whom they sent their patients, only 57% created a PMS.[14]

Incorporating training in HCT practices in pediatric graduate medical education has proven challenging. One cohort of both pediatric residents and staff pediatricians highly rated the importance of specific training (4.3 on a 5-point Likert scale) and professed their comfort level to be low (2.6 on a 5-point Likert scale), yet only 4% of participants had received formal training during residency.[5] This knowledge gap translated locally. We conducted a needs assessment in March 2018 among pediatric residents at Baylor College of Medicine-The Children's Hospital of San Antonio (BCM-CHofSA), an academic, freestanding children's hospital. The needs assessment revealed that residents were interested in learning about identifying the steps necessary for an effective HCT, becoming proficient in sharing a transition policy with families, and creating PMSs.

One previously published HCT curriculum[15] utilizes a case-based strategy over three 75- to 120-minute sessions to improve learners' self-reported comfort level with engaging in HCT practices. This curriculum can provide a much-needed initial introduction to the steps of transition to adult-oriented medical care and the roles of various health care professionals and state and local resources in supporting well-planned transitions. However, a gap remains for assessing learner skills and behaviors. Another published resource provides a case for discussion of a 15-year-old with Crohn's disease making a transition to adult care,[16] but a step-by-step process for implementing an HCT or developing a PMS is not included. Finally, Bradford and Mulroy published a resource to foster the transformation of medical students into transition coaches for YSHCN and assess knowledge and skills gained through reflection.[17] Although reflective narratives illustrate the benefits of this experience, the educational product does not create protected time for learners, which can result in diminished engagement.

The aim of our project was to design an HCT curriculum including assessment of pediatric residents' knowledge, skills, and behaviors in the transition of YSHCN. Before such a curriculum could be implemented, there first needed to be a systematic HCT process; thus, our first step was to implement a clear process within both our Pediatric Primary Care and Complex Care Clinics using tools available on the GotTransition platform, a federally funded national resource center on HCT.[18] This process included collaborating with the information technology team to incorporate the autopopulation of our HCT policy (GotTransition core element 1), development of an HCT tracking and monitoring dot phrase (GotTransition core element 2), uploading of a transition readiness assessment (GotTransition core element 3), and creation and autopopulation of a PMS (GotTransition core element 4). With a clear process in place, we then could develop and implement a curriculum to teach the needed skills. The curriculum did not address GotTransition core elements 5 and 6. Mapping of curricular elements to GotTransition core elements is illustrated in Table 1.

We believed that utilizing diverse teaching methods, allowing protected time to consume educational materials and reflect upon them, and applying newly learned skills in real time with patients/families were optimal strategies for learning. Additionally, we aimed to achieve behavioral outcomes (i.e., Kirkpatrick level 3[19]). Consequently, we integrated both synchronous and asynchronous teaching methods, afforded time to view didactic modules, and assessed learners' behavior directly in a clinical setting. Our teaching philosophy aligned with Kolb's experiential learning cycle and cognitive apprenticeship frameworks (Figure). Both frameworks asserted that learners would develop expertise in applying newly learned content through modeling, coaching, practical application, reflection, and exploration. This would allow

Copyright © 2022 Kaushik and Niebuhr. This is an open-access publication distributed under the terms of the Creative Commons Attribution license.

Table 1. Mapping of Pediatric Resident Health Care Transition Curricular Elements to GotTransition Core Elements

GotTransition Core Element	Curricular Element
1: Sharing the transition policy	DM part 3: Your Primary Care Clinic's Health Care Transition Process<br>Facilitator guide
2: Tracking and monitoring	DM part 3: Your Primary Care Clinic's Health Care Transition Process<br>Facilitator guide
3: Assessing transition readiness	DM part 3: Your Primary Care Clinic's Health Care Transition Process<br>Facilitator guide
4: Transition planning	DM part 1: Introduction<br>DM part 2: Team Transition<br>DM part 4: The Patient/Family-Centered Approach<br>DM part 5: Portable Medical Summaries and Transfer of Care<br>Facilitator guide
5: Transfer of care	Not presented
6: Transfer completion	Not presented

Abbreviation: DM, didactic module.

them to approach similar situations of increasing complexity through sequencing.

Methods

Target Learners and Setting

The target learners were pediatric residents and pediatric hospital medicine fellows at BCM-CHofSA. The curriculum was embedded within a 4-week complex care elective block rotation, which occurred within the Complex Care Clinic. This clinic was housed within the Pediatric Primary Care Clinic and served approximately 200 children/youth with medical complexity (i.e., patients depending on at least one medical/technological device and needing specialty care from at least two pediatric subspecialists). Of those with complex, chronic diagnoses, 68%

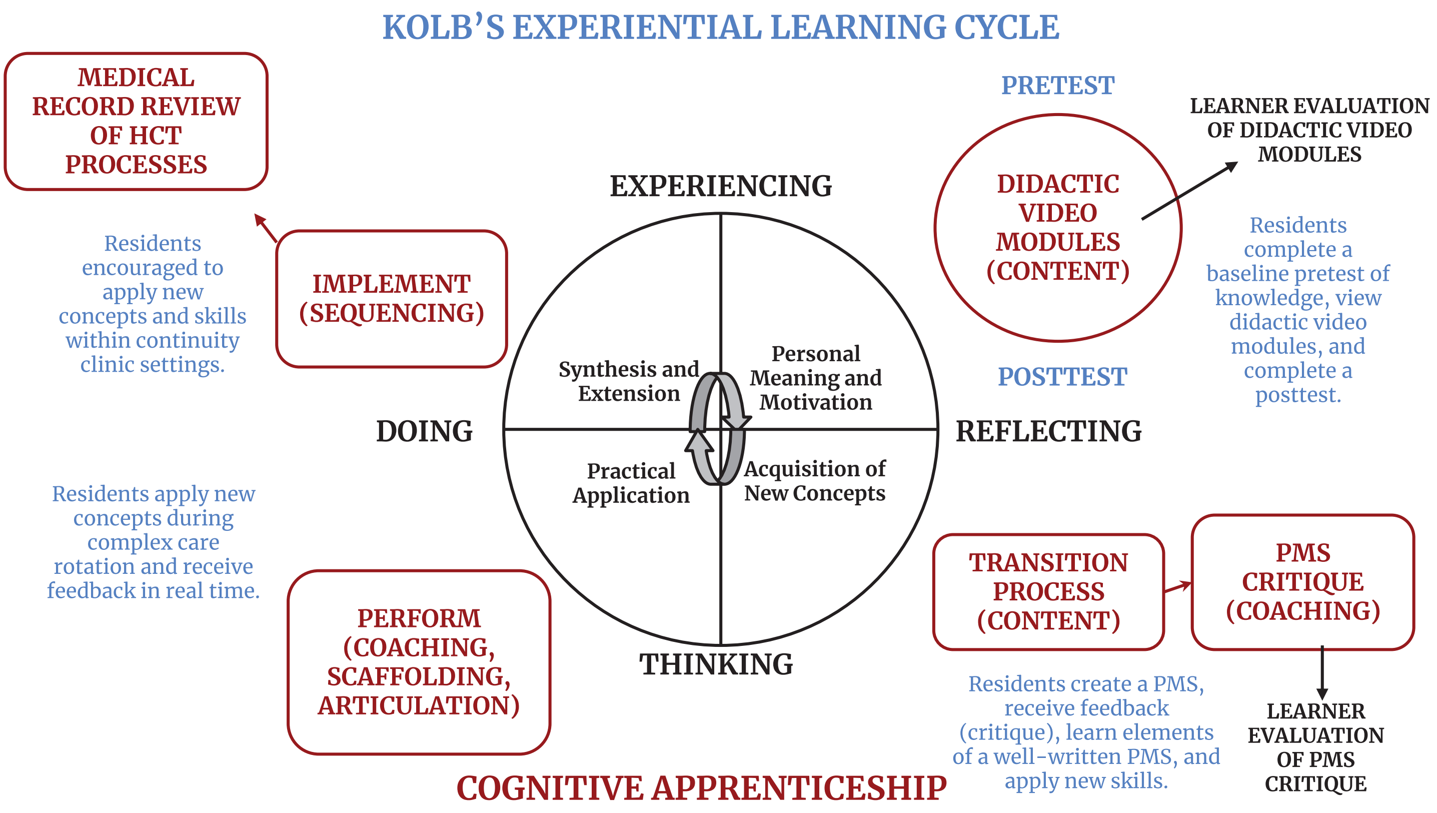

Figure. Application of Kolb's experiential learning cycle and cognitive apprenticeship frameworks to the pediatric resident HCT curriculum. Abbreviations: HCT, health care transition; PMS, portable medical summary.

Copyright © 2022 Kaushik and Niebuhr. This is an open-access publication distributed under the terms of the Creative Commons Attribution license.

received private duty nursing services, 76% had a feeding tube (nasogastric, gastrostomy, or gastrojejunostomy), 20% had a tracheostomy tube, 27% were dependent upon invasive or noninvasive mechanical ventilation, and 6% had central venous access.

Curriculum Methods

Knowledge prerotation test: Residents completed a baseline prerotation test assessing HCT knowledge, (Appendix A), best delivered using survey software.

Modules: Residents viewed five didactic modules, designed to be viewed asynchronously.

- Part 1: Introduction (Appendix B).
- Part 2: Team Transition (Appendix C).
- Part 3: Your Primary Care Clinic's HCT Process (Appendix D).
- Part 4: The Patient/Family-Centered Approach (Appendix E).
- Part 5: PMSs and Transfer of Care (Appendix F).

Parts 1, 2, 4, and 5 were concise (under 6 minutes in length) animated and narrated videos created with Powtoon software. Part 3 was a PowerPoint presentation. A facilitator guide (Appendix G) was also available to assist educators with the implementation and delivery of part 3.

Knowledge postrotation test: After viewing all modules, residents completed a postrotation test of knowledge (Appendix H), best delivered using survey software.

PMS critique exercise: During the rotation, residents created a PMS for one clinic patient, using a Word document. Residents were asked to write the PMS without searching the internet for tips on elements of a well-written PMS or reading other well-written PMSs. The rotation director critiqued each resident's PMS for inclusion of 18 elements of a well-written PMS, a list compiled from resources from GotTransition.[18] A facilitator guide to critiquing PMSs (Appendix G) was available for the rotation director's use. Following the critique, the rotation director conducted a face-to-face session with the resident to teach these 18 elements, review the resident's PMS, and demonstrate how to create a PMS within the electronic health record. The rotation director also provided the list of 18 recommended elements to residents during the PMS critique exercise. Residents then created a second PMS for the same patient, using the template within the electronic health record. The rotation director reviewed this second attempt for inclusion of the 18 elements and gave feedback to the resident.

Evaluation Methods

PMS element frequency data for both the first and second attempts were collected for each resident and tabulated in a spreadsheet. Following the didactic modules and the PMS critique exercise, residents completed 5-point Likert-scale surveys (1 = *not at all,* 5 = *extremely*) to evaluate each curriculum element (Appendices I and J, Kirkpatrick level 1). Surveys were conducted using REDCap, delivered through email.

Pre- and postrotation test scores and PMS element frequency data were presented using means and standard deviations. Both data sets were checked for deviations from normality, and differences between the two time points were evaluated using paired t tests. Statistical significance was set at $p < .05$.

Results

Between October 2019 and June 2021, 21 learners (19 pediatric residents and two pediatric hospital medicine fellows) completed the curriculum. All learners attested to having viewed the didactic modules and to writing a PMS.

Knowledge: Prerotation/Postrotation Test Results

Nineteen prerotation and postrotation tests were available for analysis. (One learner did not complete either a pretest or a posttest, and one learner did not complete a posttest.) Learners showed a statistically significant gain in knowledge (58%, SD = 0.12, vs. 94%, SD = 0.06; $p < .00001$; Kirkpatrick level 2).

Behavior: PMS Critique Exercise Results

Nineteen pre- and postcritique exercise PMSs were available for analysis. (Two learners did not submit a second attempt.) Learners included an average of eight (46%, SD = 0.10) and 17 (98%, SD = 0.04) of 18 recommended elements prior to and following the PMS critique exercise, illustrating a statistically significant ($p < .00001$) change in learner behavior (Kirkpatrick level 3).

Evaluation Results

Available for analysis were 16 didactic module evaluations and 12 PMS critique exercise evaluations.

Learners selected Likert-scale responses 4 or 5 for all didactic module evaluation questions (*effective, enjoyable, format appropriate, format ideal, likely to change*).

Thematic content analysis of learners' noted reactions to the didactic modules is illustrated in Table 2 (Kirkpatrick level 1).

On PMS critique exercise evaluations, 33% of respondents reported confidence (Likert-scale responses of 4 or 5) in their

Copyright © 2022 Kaushik and Niebuhr. This is an open-access publication distributed under the terms of the Creative Commons Attribution license.

ability to create a PMS prior to reading a list of elements of a well-written PMS, and 25% reported confidence (Likert-scale responses of 4 or 5) in their ability to create a PMS prior to completing the PMS critique exercise with the rotation director. All respondents (100%) reported confidence (Likert-scale responses of 4 or 5) in their ability to compose a PMS following the PMS critique exercise. Learners selected Likert-scale responses 4 or 5 for the remaining PMS critique exercise evaluation survey questions (*format appropriate, likely to change*).

Thematic content analysis of learners' noted reactions to the PMS critique exercise is illustrated in Table 2 (Kirkpatrick level 1).

Discussion

Our goal was to design a pediatric resident curriculum to improve HCT knowledge, skills, and behavior. We first developed a clear HCT process for our Pediatric Primary Care and Complex Care Clinics health care system by adapting nationally developed guidelines and resources. We then implemented a new HCT curriculum to teach pediatric residents the significance of a well-planned and safe HCT, particularly for YSHCN, and the practical application of HCT processes. We delivered the overwhelmingly well-received curriculum during an elective complex care 4-week block rotation, incorporating asynchronous didactic and synchronous experiential methods. We demonstrated an improvement in knowledge and skills in executing HCT processes, as well as in skills and behavior in formulating a well-written PMS, while championing self-management and self-advocacy.

We offer recommendations for other programs interested in adopting and adapting this curriculum. During the complex care rotation, our residents participated in outpatient clinical duties in the morning only, allowing them to review didactic materials in the afternoon. We believe protected time is integral to asynchronous learning. Indeed, designating the afternoon for completion of the curricular activities proved valuable, emphasized by the fact that almost all 21 learners completed pre- and posttests and viewed the didactic modules. Learners noted as strengths of this curriculum the concise nature of the modules and flexibility to watch when their schedules permitted. Moreover, learners found the asynchronous portion to be effective, engaging, and an ideal and appropriate format for learning this material.

For pediatric residency programs with no such complex care block rotation, we encourage educators to identify similar venues during which this curriculum might be used. Because the relevance of HCT processes for YSHCN translates to numerous other populations, educators may consider integrating the curriculum into other rotations where youth patients are making transitions to adult care, such as adolescent primary care, advocacy and community health, developmental-behavioral pediatrics, or any number of pediatric rotations.

Table 2. Thematic Content Analysis of Pediatric Resident/Fellow Perceptions of the Health Care Transition Curriculum

Theme	Supporting Quotes
An animated video format for delivery of health care transition didactic instruction is convenient, relevant, and enjoyable.	"Short videos… for schedule flexibility."<br>"Easy to watch, engaging."<br>"Easy to stay focused."<br>"More enjoy[able] presentation of material than a word document or pdf."<br>"Clear, logical format… synchronizes well with the activities during the rotation like creating a portable medical summary."
The didactic portion of a health care transition curriculum teaches content and processes not traditionally taught in other medical education settings.	"Focused on topics… typically not exposed to on other rotations or in medical school."<br>"Learning about various resources available to help better advocate for… patients as they transition to adulthood… [and] identifying the various steps of the transition process and their importance."
Knowledge and skills taught during a one-on-one portable medical summary critique exercise are relevant to future health care transition practice.	"Easily accessible and complete list now available for future patients."<br>"It was good to be able to go over the required points together. I did not previously know there was a function for this in [the EHR]."<br>"Helping identify information that may be important for medical providers in any setting (outpatient, inpatient, EMS) and organizing it in a concise manner. Sometimes we focus on only info we would need to know in a particular capacity but it was helpful to learn about other important things such as the preferred emergency location which also play into patient care."<br>"[Educator's] insight on what is helpful for her on the outpatient side when chronic patients are discharged from the hospital."<br>"Instant applicability with the chance to work within the framework on [the EHR]."
The instructional process of a one-on-one portable medical summary critique exercise is sensible and interactive.	"It is short but focused on the important elements of creating a portable medical summary. The template is easy to follow."<br>"Easy to follow, interactive, concise, in-person."<br>"Having the ability to try to produce a portable medical summary first and then reviewing together."

Abbreviations: EHR, electronic health record; EMS, emergency medical services.

Copyright © 2022 Kaushik and Niebuhr. This is an open-access publication distributed under the terms of the Creative Commons Attribution license.

Part 3 of the didactic modules is a PowerPoint presentation, provided here in editable format, which allows educators to individualize it for their own institution-specific HCT processes. Also, educators may choose to present part 3 synchronously. We have provided a facilitator guide (Appendix G) to assist with implementing a transition process in a health care system.

Learners found that the synchronous, interactive PMS critique exercise was an appropriate format and inspired a likelihood to change future behavior. Again, we allotted protected time to formulate PMSs, both prior to and following the critique exercise, and consequently, 17 learners submitted two PMSs for review.

A limitation of our curriculum is that it does not provide teaching about GotTransition core elements 5 (a process to identify adult health care practitioners and transfer care) and 6 (how to evaluate the clinic's transition completion). This is, by far, one of the more challenging aspects of HCT in the US,[20] particularly for health care organizations that are primarily pediatric, such as our own freestanding children's hospital, and therefore lack within-system adult provider groups to accept these patients. Educators with a transition process robust enough to address these core elements are advised to incorporate these elements into the presentation of didactic module part 3. Moreover, for institutions that have yet to incorporate an HCT process, delivering such a curriculum can prove challenging. We offer the facilitator guide with brief descriptions of our experience to assist educators initiating the planning of an HCT process.

Although our learners evaluated the curriculum quite positively, the complex care rotation was an elective block rotation, and evaluation comments reflected reactions of learners who opted to participate. Perceptions of residents who are required to complete such instructional material may vary. Nevertheless, because of the pre- and posttest completion rates and evaluation response rates, we believe all learners will appreciate the components of our product as HCT is relevant both to general pediatrics and across numerous pediatric subspecialties.

Finally, although we have assessed improvement in knowledge, skills, and behavior immediately following application of this curriculum, we have not yet followed learners longitudinally to assess whether they employ these knowledge, skills, and behavior in future practice. That sustained knowledge retention may be inadequate is evidenced by one resident recommending in the didactic module survey that we improve our discussion of methods of transition readiness assessment, initiation of the conversation, and barriers faced by patients/families. These are all topics addressed in the presentation of didactic module part 3. As we continue to deliver this curriculum, to better assess its robust and long-term impact we will consider not only retesting learners 6-12 months following the rotation for content knowledge (Kirkpatrick level 2) but also reviewing medical records for the inclusion of PMSs (Kirkpatrick level 3).

In summary, with its focus on the behavioral outcome of actually creating a PMS for transition of care, this HCT curriculum addresses a significant gap in pediatric graduate medical education. The curriculum utilizes nationally recommended concepts and guidelines and includes learning materials, assessment tools, and evaluation tools. Moreover, the curriculum is modifiable by other users to allow individual residency programs to teach in the context of their own health care systems' transition processes.

Appendices

A. Prerotation Test.docx
B. Part 1.mp4
C. Part 2.mp4
D. Part 3.pptx
E. Part 4.mp4
F. Part 5.mp4
G. Facilitator Guide.docx
H. Postrotation Test.docx
I. Didactic Module Evaluation.docx
J. Summary Critique Evaluation.docx

All appendices are peer reviewed as integral parts of the Original Publication.

Ruchi Kaushik, MD, MPH: Associate Professor, Department of Pediatrics, Baylor College of Medicine and The Children's Hospital of San Antonio; ORCID: https://orcid.org/0000-0002-6769-2801

Virginia Niebuhr, PhD: Clinical Professor, Department of Pediatrics, University of Texas Medical Branch School of Medicine/UTMB Health

Acknowledgments
We would like to acknowledge and thank Dr. Kitty O'Hare, Dr. Rita Nathawad, and Dr. Emily Goodwin for reviewing and providing suggestions during the development of the didactic modules and Dr. Alexis Wood for providing statistical support.

Disclosures
None to report.

Copyright © 2022 Kaushik and Niebuhr. This is an open-access publication distributed under the terms of the Creative Commons Attribution license.

Funding/Support
None to report.

Ethical Approval
The Baylor College of Medicine Institutional Review Board approved this project.

References

1. Cooley WC, Sagerman PJ; American Academy of Pediatrics, American Academy of Family Physicians, and American College of Physicians, Transitions Clinical Report Authoring Group. Supporting the health care transition from adolescence to adulthood in the medical home. *Pediatrics*. 2011;128(1):182-200. https://doi.org/10.1542/peds.2011-0969
2. National Survey of Children With Special Health Care Needs: NS-CSHCN 2009/10—outcome #6. Child and Adolescent Health Measurement Initiative, Data Resource Center for Child and Adolescent Health. Accessed November 10, 2019. http://www.childhealthdata.org/browse/survey/results?q=1616&r=1&r2=45
3. O'Sullivan-Oliveira J, Fernandes SM, Borges LF, Fishman LN. Transition of pediatric patients to adult care: an analysis of provider perceptions across discipline and role. *Pediatr Nurs*. 2014;40(3):113-120.
4. Wirth B, Kuznetsov A. *Shared Plan of Care: A Tool to Support Children and Youth With Special Health Care Needs and Their Families*. National Center for Medical Home Implementation; 2016. Accessed January 30, 2022. https://medicalhomeinfo.aap.org/tools-resources/Documents/Shared%20Plan%20of%20Care2.pdf
5. Weeks M, Cole B, Flake E, Roy D. Resident and staff satisfaction of pediatric graduate medical education training on transition to adult care of medically complex patients. *Mil Med*. 2018; 183(11-12):e676-e679. https://doi.org/10.1093/milmed/usy057
6. Lozano P, Houtrow A. Supporting self-management in children and adolescents with complex chronic conditions. *Pediatrics*. 2018;141(suppl 3):S233-S241. https://doi.org/10.1542/peds.2017-1284H
7. Ness MM, Saylor J, Selekman J. Maternal experiences of transitioning their emerging adult with type 1 diabetes to college. *Diabetes Educ*. 2018;44(2):178-187. https://doi.org/10.1177/0145721718759980
8. Varty M, Popejoy LL. A systematic review of transition readiness in youth with chronic disease. *West J Nurs Res*. 2020;42(7): 554-566. https://doi.org/10.1177/0193945919875470
9. Spain PD, Oeffinger KC, Candela J, McCabe M, Ma X, Tonorezos ES. Response to a treatment summary and care plan among adult survivors of pediatric and young adult cancer. *J Oncol Pract*. 2012;8(3):196-202. https://doi.org/10.1200/JOP.2011.000345
10. Chouteau WA, Allen SR. Implementation of a portable medical summary for adolescents and young adults with medical complexity in transition to adult health care. *J Pediatr Nurs*. 2019;48:35-41. https://doi.org/10.1016/j.pedn.2019.05.012
11. McLaughlin SE, Diener-West M, Indurkhya A, Rubin H, Heckmann R, Boyle MP. Improving transition from pediatric to adult cystic fibrosis care: lessons from a national survey of current practices. *Pediatrics*. 2008;121(5):e1160-e1166. https://doi.org/10.1542/peds.2007-2217
12. Garvey KC, Telo GH, Needleman JS, Forbes P, Finkelstein JA, Laffel LM. Health care transition in young adults with type 1 diabetes: perspectives of adult endocrinologists in the U.S. *Diabetes Care*. 2016;39(2):190-197. https://doi.org/10.2337/dc15-1775
13. Dale CM, King J, Amin R, et al. Health transition experiences of Canadian ventilator-assisted adolescents and their family caregivers: a qualitative interview study. *Paediatr Child Health*. 2017;22(5):277-281. https://doi.org/10.1093/pch/pxx079
14. Davidson LF, Chhabra R, Cohen HW, Lechuga C, Diaz P, Racine A. Pediatricians transitioning practices, youth with special health care needs in New York State. *Clin Pediatr (Phila)*. 2015;54(11):1051-1058. https://doi.org/10.1177/0009922815573940
15. Gooding H, O'Reilly C, Weitzman E, Burke P, Goncalves A, Pitts S. Transitioning Bryan: the transition to adulthood and the medical home. *MedEdPORTAL*. 2015;11:10226. https://doi.org/10.15766/mep_2374-8265.10226
16. Fishman L. But Tommy likes it here: moving to adult medicine. *MedEdPORTAL*. 2012;8:9190. https://doi.org/10.15766/mep_2374-8265.9190
17. Bradford N, Mulroy B. Medical students as coaches in transitions of care for youth with special health care needs. *MedEdPORTAL*. 2015;11:10183. https://doi.org/10.15766/mep_2374-8265.10183
18. GotTransition. Accessed February 17, 2022. https://www.gottransition.org
19. Macdougall CF. Evaluation—the educational context. *Arch Dis Child Educ Pract Ed*. 2010;95(1):28-32. https://doi.org/10.1136/adc.2008.142240
20. White PH, Cooley WC; Transitions Clinical Report Authoring Group; *et al*. Supporting the health care transition from adolescence to adulthood in the medical home. *Pediatrics*. 2018;142(5):e20182587. https://doi.org/10.1542/peds.2018-2587

Received: August 27, 2021
Accepted: January 16, 2022
Published: April 1, 2022

Copyright © 2022 Kaushik and Niebuhr. This is an open-access publication distributed under the terms of the Creative Commons Attribution license.
